# Mangrove Against Invasive Snails: *Aegiceras corniculatum* Shows a Molluscicidal Effect on Exotic Apple Snails (*Pomacea canaliculata*) in Mangroves

**DOI:** 10.3390/plants14050823

**Published:** 2025-03-06

**Authors:** Xinyan Yang, Hongmei Li, Huizhen Xie, Yanfang Ma, Yuting Yu, Qingping Liu, Junhao Kuang, Miaoying Zhang, Jinling Liu, Benliang Zhao

**Affiliations:** 1 College of Natural Resources and Environment, South China Agricultural University, Guangzhou 510642, China; yxy005011@stu.scau.edu.cn (X.Y.);; 2 College of Biology and Food Engineering, Guangdong University of Education, Guangzhou 510303, China

**Keywords:** *Aegiceras corniculatum*, *Pomacea canaliculata*, extracts, molluscicidal effects, hepatopancreas tissues

## Abstract

Apple snails (*Pomacea canaliculata*), one of the 100 most serious invasive species in the world, have invaded mangrove wetlands due to their salinity tolerance. We firstly prepared a plant molluscicide against apple snails based on the mangrove *Aegiceras corniculatum* in coastal wetland. The effects of four mangrove extracts from *A. corniculatum*, including ethanol extract (EE), petroleum ether extract (PEE), ethyl acetate extract (EAE), and n-butanol extract (BE), were studied for molluscicidal activity against apple snails in a saline environment. The LC_50_ values at 48 h of EE, PEE, EAE, and BE were 25 mg/L, 123 mg/L, 170 mg/L, and 14 mg/L, respectively. BE had the highest molluscicidal value (96.7%) against apple snails at 48 h. At 48 h, BE of *A. corniculatum* leaves significantly decreased the soluble sugar content, soluble protein content, acetylcholinesterase, and glutathione of apple snails to 4.25 mg/g, 29.50 mg/g, 947.1 U/gprot, and 6.22 U/gprot, respectively, compared to those in the control. The increased BE concentration significantly enhanced the malondialdehyde and aspartate aminotransferase contents to 4.18 mmol/gprot and 18.9 U/gprot at 48 h. Furthermore, the damage in the hepatopancreas tissue of apple snails increased, and the cellular structure became necrotic as the concentration of BE from *A. corniculatum* increased. The content of palmitic acid in BE of *A. corniculatum* leaves was the highest (10.9%), possibly be a toxic ingredient against apple snails. The n-butanol extract of *A. corniculatum* leaves showed a potential to control apple snails in the brackish water, and its plantation was beneficial to control the further spread of apple snails in mangrove wetlands.

## 1. Introduction

Mangrove wetlands are primarily distributed in the transitional zone between tropical and subtropical coasts [1], occupying a total area of 147,000 km^2^ of the world [2]. Asia has the largest mangrove area of over 6.8 million hm^2^ [3]. With complex community structures and rich biodiversity, mangrove wetland can improve water quality, reduce flood damage, capture carbon, and provide habitats for plants and animals, becoming an essential ecological barrier in coastal areas [4]. Mangrove wetlands are one of the most productive ecosystems in the world, providing diverse coastal ecological services [4]. However, due to the impact of human activities, there has been a significant decline in mangrove areas globally, with a total of 152,604 km^2^ of mangrove forests in 1996 and a loss of 5245 km^2^ by 2020, and an estimated further loss of 3.4% was predicted for 2024 [5].

Biological invasion brings negative changes in the structure and function of the ecosystem, resulting in serious economic losses [6,7]. Mangrove wetlands are facing the challenges of invasive species due to human activities, climate change, and the inherent complex habitat [8]. According to the Invasive Species Specialist Group (ISSG), at least 8 species of invasive plants and 10 species of invasive animals have invaded mangroves in past ten years (Table S1) [9,10,11,12,13,14,15,16,17,18,19,20,21,22,23]. Apple snail (*Pomacea canaliculata* Lamarck, 1822) (Mesogastropoda: Pilidae) is a freshwater snail native to South America. In 1981, it was introduced to China as an edible snail and rapidly spread to many places in just a few decades. After their introduction into China as an edible high-protein food in the 1980s, apple snails escaped into aquatic habitats such as paddy fields, ditches, rivers, ponds, and lakes [24]. Apple snails seriously harm freshwater wetlands, as they can feed on various macrophytes [25]. Apple snails are also an intermediate host of nematodes (*Angiostrongylus cantonensis* Chen, 1935) (Rhabditida: Strongylidae), which pose a threat to residents due to *Eosinophilic meningitis* [26,27]. Apple snails can survive in salinity water below 7.5 ppt [28]. Studies have reported that the apple snails have established populations in some coastal areas, such as the Chilean lagoon, Nansha mangrove wetlands in Guangzhou, and nature reserve mangroves on the west coast of Hainan Island, China [29,30,31].

We found that apple snails have invaded the mangrove wetlands in Guangzhou, China, and apple snails maintain their population by feeding on a large amount of mangrove leaves, especially *Acanthus ilicifolius* L. (1753) (Lamiales: Acanthaceae) [29,32]. Compared to traditional measures [33], plant-derived extracts have the potential to control apple snails due to their strong molluscicidal activity, eco-friendly characteristic, and low induced resistance [34]. Studies have reported the effect of plant-based molluscicides targeting the apple snail. For example, petroleum ether extract of *Chimonanthus nitens* (Olivier, 1887) (Laurales: Calycanthaceae) has an IC_50_ value of 0.287 mg/mL for 24 h [35]. Water extract of tobacco (*Nicotiana tabacum* L., 1753) (Solanales: Solanaceae) led to a mortality of over 90% against apple snails within 4 days [34]. However, plant-based molluscicides are mainly from freshwater plants, and little research focus on the molluscicidal properties of mangroves.

In mainland China, river mangroves *Aegiceras corniculatum* (L.) Blanco, 1753 (Ericales: Primulaceae) mainly grow in Guangdong, Guangxi, Fujian, and Hainan Provinces; in particular, Guangdong Province has the largest mangrove forests, with an area of 5169 km^2^ [36]. Mangrove always contains saponins, flavonoids, triterpenes, tannins, quinones, and other bioactive components [37], with cytotoxic and fungicidal inhibitory effects [38]. As apple snails have invaded mangrove wetlands, the development of mangrove-based molluscicides is of great value for the efficient control of apple snails in the invaded mangrove wetlands. As a typical plant in a brackish habitat invaded by apple snails, however, there is no research on the molluscicidal effect of river mangroves. We studied the molluscicidal activity of different extracts from river mangroves through toxicity measurement and chemical component analysis. We asked the following questions:

(1) Does mangrove leaf extract cause death or growth inhibition in apple snails?
(2) Does mangrove extract negatively affect the physiological and tissue characteristics of apple snails?
(3) Are there any components in mangrove extracts that are potentially molluscicidal to apple snails?

This study aims to develop new plant-based molluscicides based on mangroves in wetlands invaded by apple snails, which helps to assess the molluscicidal potential of mangroves and control apple snails in brackish water.

## 2. Results

### 2.1. Toxicity of Mangrove Leaf Extract on Apple Snails

**Figure 1:**
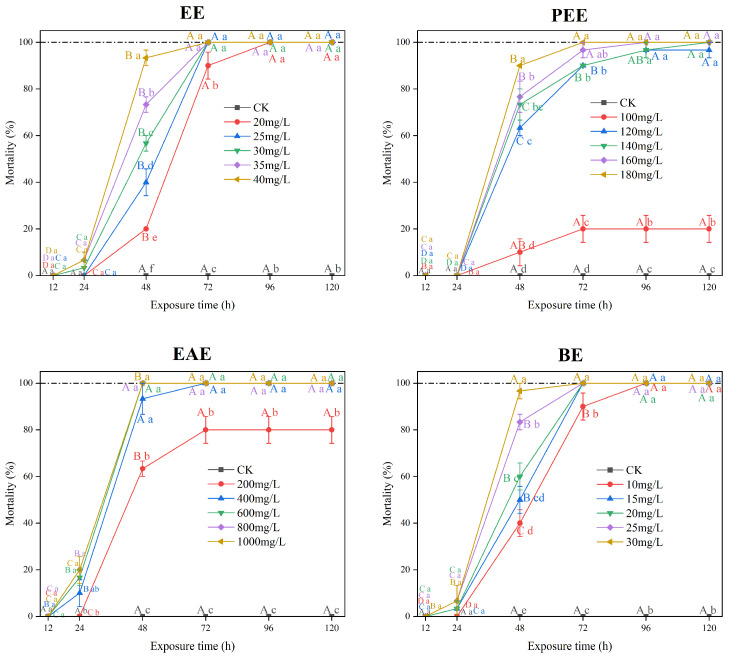
Toxic effects of leaf extracts from river mangrove (*Aegiceras corniculatum*) on apple snails. Note: EE—ethanol extract; PEE—petroleum ether extract; EAE—ethyl acetate extract; and BE—n-butanol extract. Different lowercase letters indicate significant differences between treatments and the control (CK) at the same exposure time (*p* < 0.05); different uppercase letters indicate significant differences between exposure time at the same concentration (*p* < 0.05). Error bars are standard errors (n = 3).

Four extracts of *A. corniculatum* leaves, including ethanol extract (EE), petroleum ether extract (PEE), ethyl acetate extract (EAE), and n-butanol extract (BE), showed differential molluscicidal activity against apple snails (Figure 1). The mortality of apple snails exposed to five concentrations of both EE and BE reached 100% after 96 h. The mortality of the apple snails showed an increasing trend with the exposure time for the four extracts, except the control. The critical exposure time occurred at 48 h, as the mortality of apple snails increased significantly from 24 h to 48 h (*p* < 0.05).

The mortality of apple snails exposed to 20 mg/L EE was significantly lower than that of the other concentrations at 48 h (*p* < 0.05). The mortality of the snails exposed to the 140 mg/L, 160 mg/L, and 180 mg/L concentrations of PEE reached 100% at 120 h. The mortality of apple snails exposed to 100 mg/L PEE was significantly lower than that of other concentrations from 48 h to 120 h (*p* < 0.05). The mortality of apple snails exposed to 200 mg/L EAE was significantly lower than that of other concentrations after 24 h (*p* < 0.05). At 48 h, the mortality of apple snails treated with 200 mg/L EAE was significantly lower than that of other concentrations (*p* < 0.05), and after 72 h, the mortality of the snails was 100% at all concentrations of EAE except for the concentration of 200 mg/L and the CK. At 72 h, the mortality of apple snails exposed to the lowest concentration of BE was significantly lower than that of other concentrations (*p* < 0.05), and after 96 h, the mortality of apple snails reached 100% at all five concentrations.

All the extracts were toxic to apple snail. The LC_50_ values of EE, PEE, EAE, and BE of river mangrove leaves against apple snails were 25 mg/L, 123 mg/L, 170 mg/L, and 14 mg/L at 48 h, respectively. The LC_25_, LC_50,_ and LC_75_ values of BE were lower than those of the other extracts (Table 1), indicating a maximum molluscicidal effect on apple snails. The LC_25_, LC_50,_ and LC_75_ values of EAE were significantly higher than those of PE and EE, showing a relatively weak molluscicidal effect. The molluscicidal effect of river mangrove extracts against apple snails were BE > EE > PEE > EAE. As a result, the n-butanol extracts of river mangrove leaves were used for the further identification of molluscicidal activity.

### 2.2. Effect of Mangrove Leaf Extract on Escape Ratio of Apple Snails

The escape ratio of apple snails decreased with the increase in the concentrations of BE of river mangrove at the four observed times (Table 2). After 0.5 h, the escape ratio of apple snails exceeded 30% in the 1–20 mg/L BE treatments, which was significantly higher than the control (*p* < 0.05). At 1 h, the escape ratio of apple snails decreased in the 15–30 mg/L treatments, and there were no escape behaviors of apple snails in the 25 mg/L and 30 mg/L treatments. After 6 h, apple snails had no more escape behaviors except for the 1.0 mg/mL treatment, and the escape ratio of apple snails in all treatments was significantly lower than that at 0.5 h. After 12 h, there were no escape behaviors of apple snails in all treatments (*p* < 0.05).

### 2.3. Inhibitory Effect of Aegiceras corniculatum BE on Apple Snails

**Figure 2:**
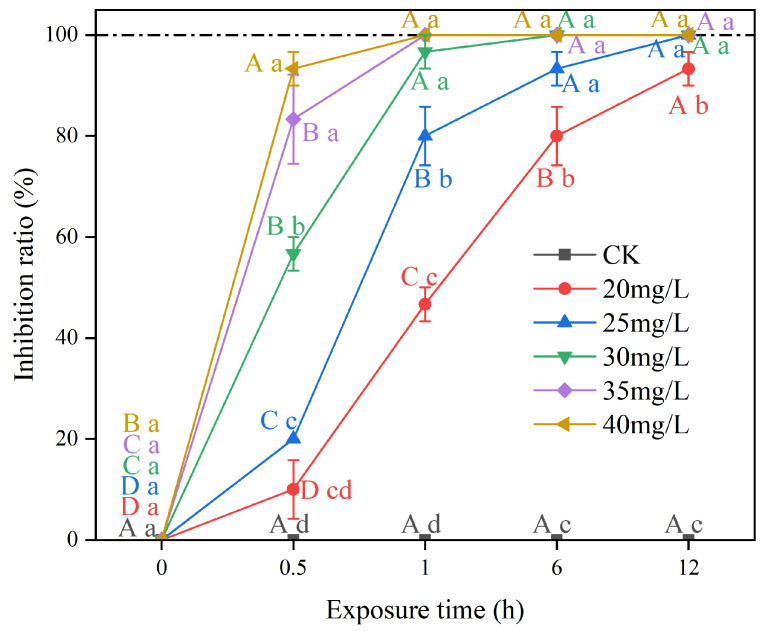
Inhibitory effect of N-butanol extract (BE) from *Aegiceras corniculatum* leaves on apple snails. Note: Different lowercase letters indicate significant differences between treatments and the CK at the same exposure time (*p* < 0.05); different uppercase letters indicate significant differences between exposure times at the same concentration (*p* < 0.05). Error bars are standard errors (n = 3).

The leaves of *Aegiceras corniculatum* BE showed a strong inhibitory effect on apple snails (Figure 2). The inhibition ratio of apple snails increased with the increase in BE concentrations at different exposure times. After 0.5 h, the inhibition ratio of apple snails in the 35 mg/L and 40 mg/L treatments exceeded 80%, significantly higher than that of other concentrations (*p* < 0.05). After 1 h, the inhibition ratio of apple snails in the 35 and 40 mg/L BE treatments reached 100%. After 6.0 h, the inhibition ratio of apple snails treated with 25–40 mg/L BE exceeded 80% after 6 h and reached 100% at 12 h. The inhibition ratio of apple snails treated with 20 mg/L BE exceeded 90% at 12 h.

### 2.4. Effect of BE on the Metabolism of Apple Snail Hepatopancreas

**Figure 3:**
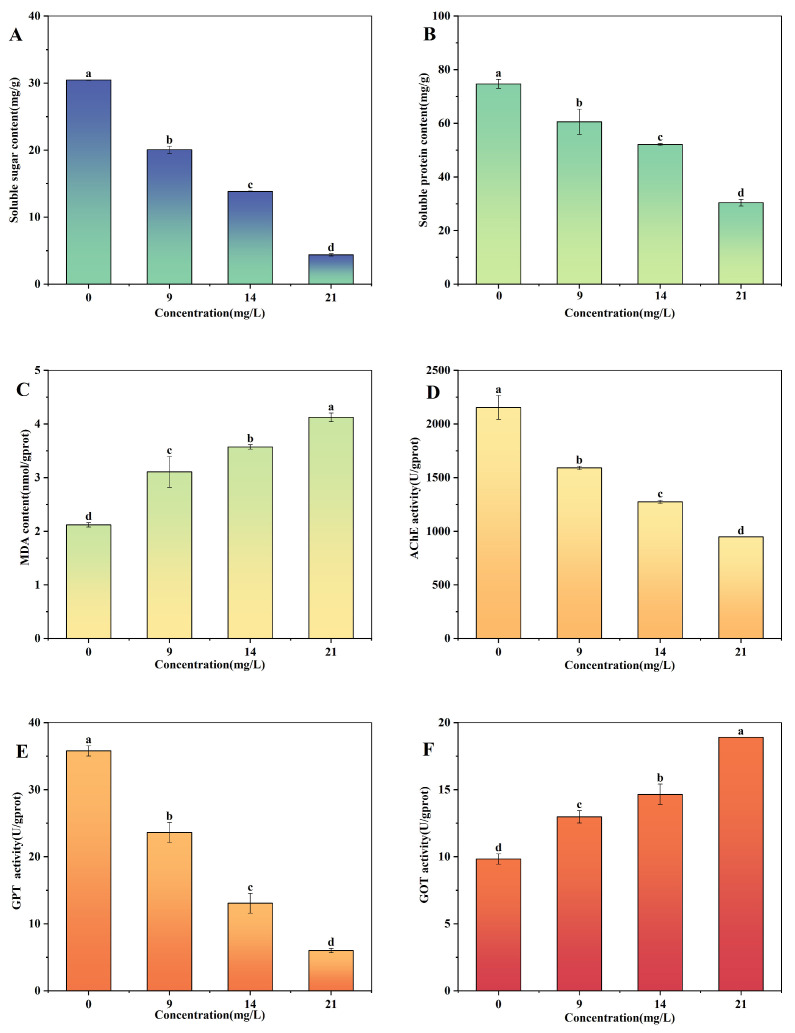
Changes in the content of soluble sugar (**A**), soluble protein (**B**), MDA (**C**), AchE (**D**), GPT (**E**), and GOT (**F**) in the hepatopancreas of apple snails exposed to n-butanol extract from *Aegiceras corniculatum* leaves. Note: Different lowercase letters indicate significant differences between treatments and the control (*p* < 0.05). Error bars are standard errors (n = 3).

The critical exposure time occurred at 48 h, as the mortality of apple snails increased significantly from 24 h to 48 h. Therefore, the effects of BE on the metabolism activity in the hepatopancreas of apple snails were assessed after 48 h of exposure. BE negatively affected the metabolism activity in the hepatopancreas of apple snails after 48 h (Figure 3). The soluble sugar content decreased significantly with an increasing concentration of BE (*p* < 0.05). The soluble sugar content in the hepatopancreas tissue of snail exposed to BE at 9 mg/L (LC_25_), 14 mg/L (LC_50_), and 21 mg/L (LC_75_) was reduced by 34%, 55% and 86%, respectively, compared to the CK (*p* < 0.05) (Figure 3A). The soluble protein contents in the hepatopancreas tissue of apple snails exposed to 9 mg/L (LC_25_), 14 mg/L (LC_50_), and 21 mg/L (LC_75_) of BE were 19%, 30%, and 59% lower than those in the CK, respectively (Figure 3B).

The MDA content of the hepatopancreas tissues of apple snails increased as the concentration of BE increased from 0 to 21 mg/L (Figure 3C). The malondialdehyde (MDA) content of the hepatopancreas tissues of apple snails exposed to 9 mg/L (LC_25_), 14 mg/L (LC_50_), and 21 mg/L (LC_75_) of BE increased by 47%, 68%, and 95%, respectively, compared with CK. Acetylcholinesterase (AchE) and glutamate transaminase (GPT) levels in the hepatopancreas tissues of apple snails exposed to 14 mg/L (LC_50_) and 21 mg/L (LC_75_) were reduced by 41% and 64% compared to the control, respectively (Figure 3E and Figure 3F). Glutamatergic aminotransferase (GOT) activity was enhanced with an increasing concentration of BE (Figure 3F). The GOT content in the hepatopancreas tissues of apple snails exposed to 14 mg/L (LC_50_) of BE increased by 33% compared to the CK.

### 2.5. Effect of BE on the Hepatopancreas Tissue of Apple Snail

**Figure 4:**
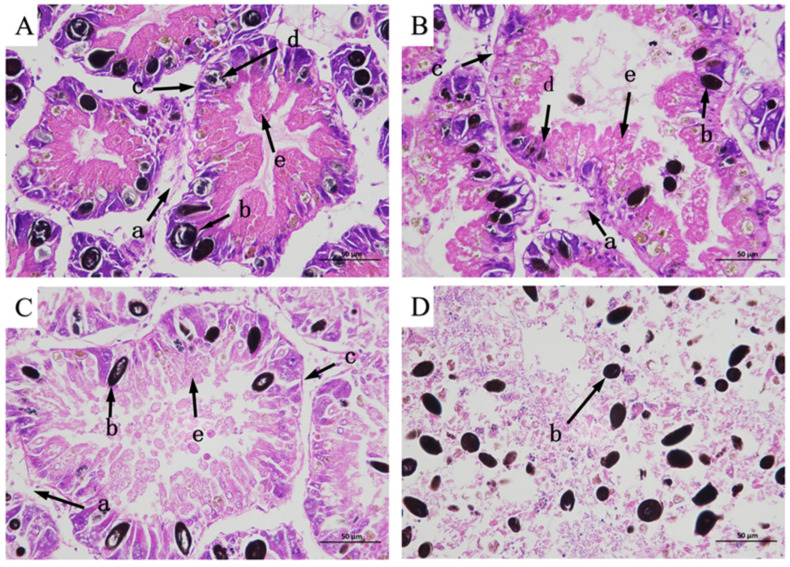
Changes in cell structure in the hepatopancreas tissue of snails exposed to 0 mg/L (**A**), 9 mg/L (**B**), 14 mg/L (**C**), and 21 mg/L (**D**) of n-butanol extract from *Aegiceras corniculatum* leaves. Note: a—connective tissue; b—black particle; c—serosa; d—basophil; and e—digestive cell.

Since the critical exposure time was determined to be 48 h, the effects of BE on the hepatopancreatic tissue of *Pomacea canaliculata* were examined after 48 h of exposure. Hematoxylin–eosin (HE) staining was performed on the hepatopancreas tissues of apple snails exposed to four concentrations of BE of *A. corniculatum* for 48 h (Figure 4). The degree of damage in the structure of the hepatopancreas tissue of apple snails increased with the increase in BE concentrations. The apple snails in the CK showed structurally intact and clear boundaries in the hepatic digestive tubules (Figure 4A). The digestive tubules were mainly composed of a large number of purple-stained digestive cells and a small number of blue-stained subrounded or narrowly elongated basophilic cells. These cells were surrounded by a uniform basement membrane, which was interspersed with black granules after staining (Figure 4A). As for apple snails exposed to 9 mg/L (LC_25_) BE, the structure of digestive tubules in the hepatopancreas tissue changed significantly compared to CK (Figure 4B), with digestive and basophilic cells undergoing a gradual decrease in lysis and membrane weakening. As for apple snails exposed to 14 mg/L (LC_50_) of BE, the digestive cells and basophils in the hepatopancreas tissue significantly decreased in solubility and dispersed; the connective tissues and the membranes tended to dissolve and become thinner (Figure 4C). The hepatopancreas tissues of apple snails exposed to 21 mg/L (LC_75_) of BE showed extensive necrosis and an incomplete structure in the digestive tubules (Figure 4D).

### 2.6. GC-MS Results of the n-Butanol Extracts

A total of 21 compounds were successfully identified in the BE of *A. corniculatum* through a GC-MS analysis (Table 3 and Figure S1), accounting for 45% of the total area of the total ion flow chromatogram. The main categories of chemical substances of BE included carboxylic acids (17.475%), alcohols (13.220%), esters (8.824%), phenols (4.253%), amino compounds (1.945%), and ketones (0.563%). The three most abundant compounds that existed in the BE after comparison with the library were n-hexadecanoic acid (palmitic acid, 10.887%), hexadecanoic acid, 2-hydroxy-1-(hydroxymethyl) ethyl ester (2-monopalmitic glycerol, 6.593%), and 1-Hexanol, 2-ethyl-(2-ethyl-1-hexanol, 4.693%).

## 3. Discussion

### 3.1. Molluscicidal Effect of Extracts of A. corniculatum Leaves

In order to explore the toxicological potential of river mangrove (*A. corniculatum*) against invasive animals, ethanol extract, petroleum ether extract, ethyl acetate extract, and n-butanol extract of river mangrove leaves were subjected to a molluscicidal assay. Four extracts of river mangrove leaves were toxic to apple snails. Previous studies have shown that extracts of mangrove plants exhibit significant pharmacological effects against animal pathogens [39,40]. River mangrove, as a medicinally important plant in the mangrove ecosystem, contains a large number of secondary metabolites in its extracts [41]. The polarity of n-butanol is relatively moderate. It is less polar than ethanol but more polar than many non-polar solvents [42]. The polarity of n-butanol enables it to dissolve both some polar compounds (such as certain sugars, amino acids, phenols, etc.) and some non-polar substances like liposoluble compounds [43,44,45]. Therefore, as a solvent, n-butanol can extract a wider range of substances. This may explain why the n-butanol extract exhibited the highest molluscicidal activity in this study. The strong activity of the n-butanol extract of river mangrove may be related to the characteristics of the secondary metabolites it contains. Plants contain various important secondary metabolites, which have exhibited significant cellular toxicity in studies (Table 4). In particular, some secondary metabolites, such as saponins and flavonoids, can damage red blood cells and disrupt the hemolytic system of mollusks, prevent respiratory processes, and inhibit metabolic activity [46]. Therefore, the secondary metabolite components in the extract of river mangrove may play an important role in the molluscicidal process. The n-butanol extract of mangrove leaves exhibited significant molluscicidal activity, surpassing the activity of extracts from the aerial parts of the invasive plant *Solidago canadensis* and certain terrestrial plants (Table 4) [47]. This could be attributed to the unique growth environment of mangrove plants, which facilitates the accumulation of a higher concentration of secondary metabolites [48].

### 3.2. Changes in Physiological and Biochemical Indices in Hepatopancreas Tissue

The hepatopancreas plays an indispensable function in detoxification and metabolic processes in apple snails [35]. The hepatopancreas is often used to detect the effects of toxicants on the overall structure due to it having first contact with intestinal exogenous substances [53,54]. In this study, a high concentration of n-butanol extract of river mangrove led to a high injury level of the hepatopancreas tissue of apple snail. This study found that the content of soluble sugars in the hepatopancreas tissue of apple snails decreased significantly with the increasing concentration of n-butanol extract. An incomplete structure of the hepatopancreas tissue affected its regulatory function for soluble sugars, leading to a malfunction in sugar metabolism and a lower content of soluble sugar [55]. Soluble sugar content in the hepatopancreas tissue of snail decreased with increasing concentration after exposure to vegetative waxberry for 48 h [56]. As for the decrease in protein content, it was possibly related to the increased demand of energy in apple snail in response to toxicity stress from the n-butanol extract of river mangrove. When glucose is depleted to a critical level, apple snails may initiate the proteolytic pathway to supply additional energy in order to maintain a balanced state of energy metabolism [57].

Malondialdehyde (MDA) is one of the main bioindicators of lipid peroxidation, reflecting the degree of lipid peroxidation and cell damage in cells and tissues [58,59]. In this study, the MDA content in the hepatopancreas tissue of apple snails increased with increasing concentration after the exposure, which was consistent with the changes in MDA content in the hepatopancreas of the snails treated with the petroleum ether extract from *Solidago canadensis* L. (1753) (Asterales: Asteraceae) [47]. HE staining of hepatopancreas tissues showed that the basement membrane outside the digestive tubules thinned until it disappeared with increasing extract concentration, indicating that the degree of oxidative damage to the cell membranes was enhanced with the increase in extract concentration.

Acetylcholinesterase (AchE) has the function of degrading acetylcholine (ACh) and terminating neurotransmission [60]. Here, AchE activity in the hepatopancreas of apple snails was significantly decreased compared with the control after exposure for 48 h. Inhibition of AChE results in the accumulation of acetylcholine, hyperpolarization of postsynaptic membranes, and interruption of nervous transmission. Inhibition of AChE can give rise to an important dysfunction, such as behavioral changes, paralysis, and death in marine organisms [61].

Glutamate aminotransferase (GOT) and glutamate transaminase (GPT) are important components of protein metabolism in animals and serve as two key aminotransferases in the mitochondria of the hepatopancreas [62]. The damaged hepatopancreas releases transaminases into the hemolymph, and therefore GOT and GPT are used as biomarkers for hepatopancreatic injury and dysfunction [63,64]. Here, the activity of GPT significantly decreased while the GOT activity significantly increased in the hepatopancreas as the concentration of the n-butanol extract of river mangrove leaves increased. A previous study found that the activities of both GPT and GOT in the hepatopancreas of apple snails increased significantly after exposure to petroleum ether extract (PEE) from *Chimonanthus praecox* (L.) Link (1777) (Magnoliales: Calycanthaceae) due to damage or necrosis of hepatopancreatic cells [35]. The reduction in GPT activity in the hepatopancreas of *P. canaliculata* caused by the n-butanol extract of mangrove leaves may be due to the high toxicity exceeding the snail’s tolerance threshold.

We think that the toxic substances present in the n-butanol extract led to the death of snails after causing hepatopancreatic tissue damage and metabolic disorders. The extract interferes with key physiological processes such as protein metabolism and enzyme activity, and the cell structure and metabolic pathways are severely damaged, ultimately leading to the death of the snail.

### 3.3. Compositional Analysis of n-Butanol Extract of Aegiceras Corniculatum Leaves

Among the 21 compounds identified from the n-butanol extract of river mangrove leaves, the 3 compounds with the highest content were n-hexadecanoic acid (palmitic acid), (hydroxymethyl)ethyl ester 2-Palmitoylglycerol (2-monopalmitic glycerol), and 1-Hexanol, 2-ethyl-(2-ethyl-1-hexanol). Components such as 3,7,11,15-tetramethyl-2-en-1-hexadecanol, palmitic acid, methyl 9,12,15-octadecadienoate, 1,2-benzenedicarboxylic acid bis (2-ethylhexyl ester), and 8,11,14-en-eicosanoic acid were isolated from the molluscicidal extracts of *S. sebiferum* leaves, and palmitic acid was deduced to be one of the active substances against snails in *S. sebiferum* [65]. Palmitic acid is the most abundant saturated fatty acid, which can enhance glucolipotoxicity and cell apoptosis. High levels of palmitic acid inhibit the activity of glucose metabolism enzymes such as glycolytic enzymes, hexokinase, and G-6-P-dehydrogenase, reducing the glucose oxidation pathway [66]. Palmitic acid can also cause lipotoxicity in hepatocytes, affecting lipid homeostasis, endoplasmic reticulum stress, and apoptosis [67]. Palmitic acid can promote the generation of free radicals by increasing the oxidation reaction of fatty acids, especially in high-metabolism organs such as the liver [68]. Hepatopancreatic homeostasis is disrupted when the quantity of free radicals, especially reactive oxygen species, exceeds the endogenous antioxidant components. Excessive reactive oxygen species in hepatocytes cause damage to proteins, lipids, and DNA, leading to structural and functional abnormalities in the liver [69]. This is combined with the fact that palmitic acid produces toxic effects on hepatocytes, induces damage to mitochondria, and inhibits protein synthesis, which leads to apoptosis [65]. Therefore, we thought that palmitic acid was the main molluscicidal component in the n-butanol extract of river mangrove leaves. Therefore, due to the abundance and diversity of benthic mollusks in mangrove wetlands, it is essential to consider the effects of mangrove extracts on non-target native mollusks. This evaluation can help assess their practical application in the field and further refine the technical parameters for optimal use.

## 4. Materials and Methods

### 4.1. Material

The leaves of river mangrove (*Aegiceras corniculatum* L. Blanco) were collected from the mangrove wetland in Nansha District, Guangzhou, China (22°62′ N, 113°66′ E, 5 m). The mean air temperature and the annual precipitation in the sampling region in 2022 were 23.4 °C and 1748.9 mm [70]. The tested individuals were the progeny of apple snails collected from the mangrove wetland in Guangzhou, China, which were previously identified as *Pomacea canaliculata* (Lamark) based on COI [29].

### 4.2. Preparation of Mangrove Leaves Extract

Fresh mangrove leaves were cleaned with pure water at least three times. After removing the surface water using filter paper, the mangrove leaves were dried at 105 °C for 2 h and then at 80 °C for 48 h in an oven (DHG-9070A, Yiheng Scientific Instrument Co., Shanghai, China) to obtain a constant weight. The dry leaves of mangroves were ground and sieved through a 60-mesh sieve. The obtained powder was stored in a dryer at room temperature (25 ± 1 °C) for further use.

The powder (200 g) of river mangrove leaves was soaked in 70% ethanol at room temperature in a ratio of 1:20 (*w*:*v*) for 24 h. The solution was subjected to ultrasonic treatment for 30 min using an ultrasonic instrument (VGT-2120QT, GT Ultrasonic Limited Co., Guangzhou, China) and then filtered to obtain the filtrate and solid residue using a vacuum pump (YR-PTB, Gongyi Yuhua Instrument Co., Shanghai, China). The solid residue was extracted three times following the above steps. The resultant filtrate from three lots of extraction was combined together. The crude extract was concentrated at 45 °C for 96 h to obtain a paste of ethanol extract using a rotary evaporator (Shanghai Yarong Biochemical Instrument Factory, Shanghai, China). The final paste of ethanol extract was then dried at 45 °C for 120 h to obtain a constant weight in an oven.

After sonicating for 1 min at room temperature (25 ± 1 °C), the final paste was further extracted with petroleum ether, ethyl acetate, and n-butanol in a ratio of 3:1 (*v*:*v*) 6 times [29,47]. The petroleum ether, ethyl acetate, and n-butanol extract were concentrated at 45 °C, 60 °C, and 55 °C for 96, 120, and 96 h, respectively, using a rotary evaporator. The resultant extract was dried at 45 °C for 120 h to obtain petroleum ether extract, ethyl acetate extract, and n-butanol extract, respectively, in an oven.

### 4.3. Determination of Mangrove Extract Toxicity Against Apple Snails

The toxicity of mangrove leaf extract on apple snails was assessed under a saline environment (2.5‰) [71]. Stock solutions of mangrove leaf extract, including ethanol extract (EE), petroleum ether extract (PEE), ethyl acetate extract (EAE), and n-butanol extract (BE), were prepared for further experiments.

Saline solution (2.5‰) without any extract was used as the control (CK). Ten healthy and active snails were placed in a plastic box (500 mL) with 400 mL of solution, which was sealed with a gauze to prevent the snails from escaping [19]. Healthy snails had a smooth and intact shell, and they moved actively in the aquaria. Adult snails with a shell height (18–25 mm) were used for the experiment. The sex ratio of apple snails was 1:1 [72]. Three replicates were used for the treatments and control.

Based on the toxicity of the four mangrove leaf extracts against apple snails in the pre-experiment, the molluscicidal characteristics of each mangrove extract against apple snails were measured in the corresponding five concentrations (Table S2). As a result, the n-butanol extracts of river mangrove leaves were used for the further identification of molluscicidal activity. Concentrations of n-butanol extracts (0, 10, 15, 20, 25, and 30 mg/L) of river mangrove were used to study the weight, inhibition effect, behavior response, and tissue changes of apple snails in the experiment. The weight, inhibition ratio, escape ratio, and mortality of apple snails were determined. The weights of apple snails at 7 d were determined using a scale (0.0001 g, Youke Instrumentation Co., Ltd., Shanghai, China) after removing the residual water on the shell and operculum with filter paper. Apple snails in the states of dead, inhibited, and escape were identified carefully (Table 5). All apple snails were checked and the mortality was recorded at 12, 24, 48, 72, 96, and 120 h [73]. The escape, inhibition, and dead behaviors of snails at different concentrations were recorded at 0.5, 1, 6, 12, and 24 h. The inhibition ratio, mortality, and escape ratio of the n-butanol extract of the river mangrove leaf on apple snails were calculated using Equations (1)–(3).

Mortality = number of dead snails/total number of test snails × 100%,(1)

Inhibition rate = number of inhibited snails/total number of test snails × 100%,(2)

Escape rate = number of escaped snails/total number of snails × 100%.(3)

### 4.4. Determination of Physiological Indicators in the Tissue

Based on the obtained LC_50_ values, three concentrations of the n-butanol extract of river mangrove, including 9 mg/L (LC_25_), 14 mg/L (LC_50_), and 21 mg/L (LC_75_), were used to determine the physiological responses of apple snails. The saline pure water without mangrove extract was the control (CK, 2.5%). Ten apple snails were placed in each plastic box.

The apple snails in the treatments and CK were cracked and the retrieved hepatopancreas tissues were used to determine the physiological characteristics at 48 h, including soluble sugar, protein, malondialdehyde (MDA), acetylcholinesterase (AchE), glutamic pyruvate transaminase (GPT), and glutamic oxalic transaminase (GOT). The hepatopancreas tissues of apple snails were first kept in a 4 °C solution, and were then ground into 10% homogenate with 0.9% physiological saline in a ratio of 1:9 (*w*:*v*). The resultant mixture was centrifuged at 4 °C, 4000 r/min for 10 min using a centrifuge (3-20R Kecheng Instrument Co., Changsha, China). The resultant supernatant was used for physiological analysis [75].

The content of soluble sugars (mg/g) was determined using the anthrone sulfuric acid colorimetric method [76]. The content of soluble protein (mg/g) was determined using the Coomassie brilliant blue method [77]. The content of malondialdehyde (MDA) and acetylcholinesterase (AchE) was determined using the thiobarbituric acid (TBA) method with MDA measurement kits (AKFA013C, AKFA005C, Box Sangong Technology Co., Beijing, China). The content of acetylcholinesterase (AchE) was determined using the DTNB method with the AchE measurement kits (AKFA005C, Box Sangong Technology Co., Beijing, China). The content of glutamic pyruvate transaminase (GPT) was determined using UV colorimetry with a GPT measurement kit. (C009-3-1, C010-3-1, Jiancheng Biological Engineering Research Institute Co., Nanjing, China). The content of glutamic oxalic transaminase (GOT) was determined using the UV colorimetry method with a GOT measurement kit (C009-3-1, C010-3-1, Jiancheng Biological Engineering Research Institute Co., Nanjing, China).

### 4.5. Observation of Hepatopancreas Tissue Structure

The hepatopancreas tissue structure of apple snails at 48 h in the treatments and CK was determined using an HE staining method [78]. The hepatopancreas tissues of apple snails were kept in a pre-cooled saline solution (4 °C, 0.9%) before determination. After removing the residual water on the shell and operculum of the apple snails using filter papers, the hepatopancreas tissues were first fixed using 4% paraformaldehyde for 24 h. Gradient dehydration of the hepatopancreas tissue was then carried out using ethanol in a gradient of 50%, 60%, 70%, 80%, and 90%. Paraffin embedding was processed using an embedding machine (YD-6D, Yidi Medical Appliance Co., Ltd., Jinhua, China), and then the samples were cooled and cured on a freezer table at −20 °C. After cooling, tissue sectioning, spreading, and baking operations were performed. The paraffin sections were first dewaxed and hydrated with water. Subsequently, the nuclei were stained with hematoxylin, followed by cytoplasmic staining with eosin. After staining, the sections underwent dehydration and were sealed with neutral resin. Finally, the HE-stained sections were examined and photographed using a Nikon light microscope [78].

### 4.6. Component Detection Using GC-MS

The composition of the n-butanol extract of river mangrove leaves was analyzed by GC-MS (GCMS-Qp2010 Uitra, Shimadzu Corporation, Kyoto, Japan) [79]. A TG-5MS column (30 m × 250 μm × 0.25 μm) was utilized for the analysis. The oven temperature was initially set at 80 °C and maintained for 2 min, followed by a temperature ramp from 80 °C to 150 °C at a rate of 8 °C per minute, and subsequently to 280 °C at a rate of 4 °C per minute. The injection volume was 1.0 μL, with helium as the carrier gas at a split ratio of 1:1. The temperatures of both the injector and detector were maintained at 280 °C.

### 4.7. Statistical Analyses

The data were sorted using Microsoft Excel 2021 (Microsoft Corp., Redmond, WA, USA). Analysis of variance was conducted using SPSS 25.0 (IBM Corp., Armonk, NY, USA), with a significance level of *p* < 0.05 or 0.01. A one-way analysis of variance (ANOVA) was used to test for significant differences between the groups, followed by post hoc tests to assess pairwise comparisons [80]. Lethal concentrations (LC_50_, LC_25_, and LC_75_) of different extracts of mangrove leaves were calculated using SPSS 25.0 through the probit module of probability unit regression [81]. The data plots were produced using Origin Pro 2021b (OriginLab Corp., Northampton, MA, USA).

## 5. Conclusions

Invasive apple snails have established populations in mangrove wetlands; it is necessary to develop new measures to control their further expansion under the brackish environment. In this study, ethanol extracts, petroleum ether extracts, ethyl acetate extracts, and n-butanol extracts of river mangrove (*Aegiceras corniculatum*) all showed toxicological activity against apple snail, among which n-butanol extracts had the strongest toxic effect. The n-butanol extract of river mangrove leaves has the potential to become a plant-derived molluscicide. The n-butanol extract of river mangrove leaves negatively affected the soluble sugar content, soluble protein content, acetylcholinesterase activity, and glutamine transaminase activity in the hepatopancreas tissue of apple snail. Furthermore, apple snail showed extensive necrosis and severe damage to cellular structure in the hepatopancreas tissue in the treatment with the n-butanol extract of river mangrove leaves. The tissues of apple snail responded significantly to the stress from the mangrove extract at the physiological and structural levels. The n-butanol extract of river mangrove leaves had the highest content of palmitic acid (10.89%), which may be the active ingredient responsible for the toxicological effect. Considering that diverse benthic animals exist in the same habitat as the apple snails in mangrove wetlands, the environmental safety of the n-butanol extract of river mangrove leaves needs to be investigated. In further research, the safety assessment of mangrove leaf extract on non-target aquatic organisms can provide a scientific basis for the rational application of plant-derived molluscicides.

## Figures and Tables

**Table 1:** Lethal concentration of different extracts of *Aegiceras corniculatum* leaves on apple snail of 48 h.

Extract	Lethal Concentrations					
	LC_25_ (mg/L)	95% Confidence Interval	LC_50_ (mg/L)	95% Confidence Interval	LC_75_ (mg/L)	95% Confidence Interval
EE	22	19–24	27	25–29	34	31–38
PEE	103	89–112	123	114–131	148	139–161
EAE	121	56–163	170	106–212	241	188–295
BE	9	6–11	14	11–16	21	19–26

Note: EE—ethanol extract; PEE—petroleum ether extract; EAE—ethyl acetate extract; and BE—n-butanol extract.

**Table 2:** The escape ratio of apple snails exposed to n-butanol extracts of *Aegiceras corniculatum*.

Concentration (mg/L)	Time (h)			
	0.5	1	6	12
0 (Control)	0 Ac	0 Ab	0 Ab	0 Aa
1.0	43.33 ± 8.82 Aa	46.67 ± 8.82 Aa	16.67 ± 8.82 Ba	0 Ba
15	53.33 ± 3.33 Aa	16.67 ± 8.82 Bb	0 Cb	0 Ca
20	33.33 ± 8.82 Aab	3.33 ± 3.33 Bb	0 Bb	0 Ba
25	13.33 ± 6.67 Abc	0 Bb	0 Bb	0 Ba
30	6.67 ± 3.33 Ac	0 Bb	0 Bb	0 Ba

Note: Different lowercase letters indicate significant differences between treatments and the control at the same exposure time (*p* < 0.05); different uppercase letters indicate significant differences between exposure times at the same concentration (*p* < 0.05). Error bars are standard errors (n = 3).

**Table 3:** Chemical constituents of n-butyl alcohol extract from *Aegiceras corniculatum*.

No.	Compound Name	Chemical Formula	Molecular Weight (amu)	Relative Content %	Compound Structure	CAS No.
1	1-Hexanol, 2-ethyl-	C_8_H_18_O	130	4.696	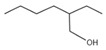	000104-76-7
2	Benzyl alcohol	C_7_H_8_O	108	0.364	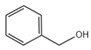	000100-51-6
3	Phenol, 2-methoxy- Guaiacol	C_7_H_8_O_2_	124	1.201	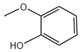	000090-05-1
4	Dibutoxy (dimethyl)silane	C_10_H_24_O_2_Si	204	0.816	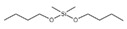	1000334-08-2
5	2-Methoxy-4-vinylphenol	C_9_H_10_O_2_	150	1.189	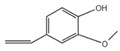	007786-61-0
6	Phenol, 2,6-dimethoxy-	C_8_H_10_O_3_	154	1.503	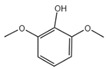	000091-10-1
7	1-(3,6,6-Trimethyl-1,6,7,7a-tetrahydrocyclopenta[c]pyran-1-yl) ethanone	C_13_H_18_O_2_	206	0.563	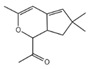	1000194-97-2
8	1,2,3-Benzenetriol	C_6_H_6_O_3_	126	0.361	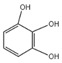	000087-66-1
9	4-((1E)-3-Hydroxy-1-propenyl)-2-methoxyphenol	C_10_H_12_O_3_	180	1.902	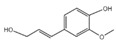	1000297-95-5
10	n-Hexadecanoic acid	C_16_H_32_O_2_	256	10.887	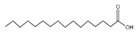	000057-10-3
11	9,12-Octadecadienoic acid (Z, Z)- Linoleic acid	C_18_H_32_O_2_	280	1.059	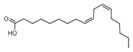	000060-33-3
12	9,12,15-Octadecatrienoic acid, (Z, Z, Z)- Linolenic acid	C_18_H_30_O_2_	278	3.949	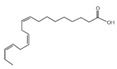	000463-40-1
13	Octadecanoic acid Stearic acid-1-13C	C_18_H_36_O_2_	284	0.595	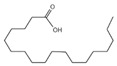	000057-11-4
14	Hexadecanoic acid, butyl ester Butyl palmitate	C_20_H_40_O_2_	313	0.985	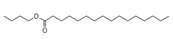	000111-06-8
15	Benzyl beta-d-glucoside	C_13_H_18_O_6_	270	1.931	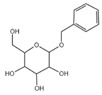	1000126-94-6
16	12-Methyl-E, E-2,13-octadecadien-1-ol	C_19_H_36_O	280	0.256	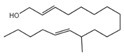	1000130-90-4
17	9- Octadecenamide, (Z)- Octadec-9-enamide	C_18_H_35_NO	281	1.945	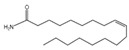	000301-02-0
18	Hexadecanoic acid, 2-hydroxy-1-(hydroxymethyl)ethyl ester 2-Palmitoylglycerol	C_19_H_38_O_4_	331	6.593	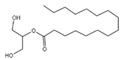	023470-00-0
19	Bis(2-ethylhexyl) phthalate Dioctyl phthalate	C_24_H_38_O_4_	391	0.460	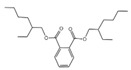	000117-81-7
20	(R)-(-)-14-Methyl-8-hexadecyn-1-ol	C_17_H_32_O	252	2.140	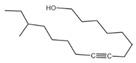	064566-18-3
21	7,10,13-Hexadecatrienoic acid, methyl ester	C_17_H_28_O_2_	264	1.772	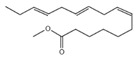	056554-30-4
Total	-	-	-	45.167	-	-

**Table 4:** Molluscicidal effect of different plant extracts on apple snail.

Plant Part	Latin Name	Extraction Type	Compound Class	Result	Citation
Chrysanthemum leaves	*Tithonia diversifolia*	methanol	alkaloids, saponins	LC_50_ = 6000 ppm LC_50_ = 3000 ppm After 24 h	[49]
Bile stem of green bovine	*Tinospora crispa*	methanol	Triterpenoid Saponin	LC_50_ = 1825 ppm After 48 h	[50]
Aralia root	*Aralia armata*	ethanol	saponin	LC_50_ = 7.90~17.50 mg/L	[51]
Mountain wax plum blossom	*Chimonanthus nitens*	petroleum ether	alkaloids, flavonoids	LC_50_ = 247 mg/L After 48 h	[35]
Glasses bean bark	*Entada rheedii*	methanol	saponins, tannins and terpenoids	LC_50_ = 1611 ppm After 72 h	[46]
Chimonanthus	*C. praecox*	petroleum ether	terpenoids	LC_50_ = 190 mg/L After 48 h	[52]

**Table 5:** Characteristics of inhibition and escape status of *Pomacea canaliculata*.

Judgment Indicator	Status	Reference
Operculum open and/or gastropods out, no response to stimulation	Dead	[74]
Sinking or floating on the water surface, operculum closed, sluggish response to pinprick stimulation	Inhibited	
Submerged or floating on the surface, ventral peduncle outside, sluggish response to pinprick stimuli	Inhibited	
Disengaged from the water surface, attached to the pelvic wall	Escape	

## Data Availability

https://figshare.com/s/b09f03f3404c570672bc (accessed on 17 January 2025).

## Supplementary Materials

The following supporting information can be downloaded at https://www.mdpi.com/article/10.3390/plants14050823/s1: Figure S1. Total ion current diagram of n-butyl alcohol extract of *Aegiceras corniculatum.* Table S1. Invasive organisms reported in mangrove wetlands. Table S2. Concentrations of mangrove extracts used in the molluscicidal test.

## References

[1] Sievers M., Brown C.J., McGowan J., Turschwell M.P., Buelow C.A., Holgate B., Pearson R.M., Adame M.F., Andradi-Brown D.A., Arnell A. (2023). Co-occurrence of biodiversity, carbon storage, coastal protection, and fish and invertebrate production to inform global mangrove conservation planning. Sci. Total Environ..

[2] Kumar A., Anju T., Archa V., Warrier V.P., Kumar S., Goud G.S., Kashyap A.K., Singh S., Komal, Singh P. (2021). Mangrove Forests. Wetlands Conservation.

[3] Jia M., Wang Z., Mao D., Ren C., Song K., Zhao C., Wang C., Xiao X., Wang Y. (2023). Mapping global distribution of mangrove forests at 10-m resolution. Sci. Bull..

[4] Akram H., Hussain S., Mazumdar P., Chua K.O., Butt T.E., Harikrishna J.A. (2023). Mangrove Health: A Review of Functions, Threats, and Challenges Associated with Mangrove Management Practices. Forests.

[5] Bunting P., Rosenqvist A., Hilarides L., Lucas R.M., Thomas N., Tadono T., Worthington T.A., Spalding M., Murray N.J., Rebelo L.-M. (2022). Global Mangrove Extent Change 1996–2020: Global Mangrove Watch Version 3.0. Remote Sens..

[6] Henry M., Leung B., Cuthbert R.N., Bodey T.W., Ahmed D.A., Angulo E., Balzani P., Briski E., Courchamp F., Hulme P.E. (2023). Unveiling the hidden economic toll of biological invasions in the European Union. Environ. Sci. Eur..

[7] Jhariya M.K., Banerjee A., Raj A., Meena R.S., Khan N., Kumar S., Bargali S.S., Jhariya M.K., Meena R.S., Banerjee A., Meena S.N. (2022). Chapter 24—Species invasion and ecological risk. Natural Resources Conservation and Advances for Sustainability.

[8] Rull V. (2022). Responses of Caribbean Mangroves to Quaternary Climatic, Eustatic, and Anthropogenic Drivers of Ecological Change: A Review. Plants.

[9] de Lima Freires J., Lage-Pinto F., Bernini E. (2023). Estimation of the extent of defoliation in *Avicennia* L. (Acanthaceae) caused by caterpillars of *Hyblaea puera* (Cramer, 1777) in a tropical mangrove. Reg. Stud. Mar. Sci..

[10] Garside C., Bishop M. (2014). The distribution of the European shore crab, *Carcinus maenas*, with respect to mangrove forests in southeastern Australia. J. Exp. Mar. Biol. Ecol..

[11] Dineen J.F., Clark P.F., Hines A.H., Reed S.A., Walton H.P. (2001). Life History, Larval Description, and Natural History of *Charybdis Hellerii* (Decapoda, Brachyura, Portunidae), an Invasive Crab in the Western Atlantic. J. Crustac. Biol..

[12] Bishop M.J., Krassoi F., Mcpherson R.G., Brown K.R., Summerhayes S.A., Wilkie E.M., O’Connor W.A. (2010). Change in wild-oyster assemblages of Port Stephens, NSW, Australia, since commencement of non-native Pacific oyster (*Crassostrea gigas*) aquaculture. Mar. Freshw. Res..

[13] Kennedy J.G.C., Johnson S.A., Brewer J.S., Leary C.J. (2021). The potential role of reproductive interference in the decline of native green treefrogs following Cuban treefrog invasions. Biol. Invasions.

[14] Silva-Oliveira G., Ready J., Iketani G., Bastos S., Gomes G., Sampaio I., Maciel C. (2011). The invasive status of *Macrobrachium rosenbergii* (De Man, 1879) in Northern Brazil, with an estimation of areas at risk globally. Aquat. Invasions.

[15] Russell D., Thuesen P., Small F. (2012). Reproductive strategies of two invasive tilapia species *Oreochromis mossambicus* and *Tilapia mariae* in northern Australia. J. Fish Biol..

[16] Burgos-Rodríguez J.A., Avilés-Rodríguez K.J., Kolbe J.J. (2016). Effects of invasive Green Iguanas (*Iguana iguana*) on seed germination and seed dispersal potential in southeastern Puerto Rico. Biol. Invasions.

[17] Sancho G., Kingsley-Smith P.R., Morris J., Toline C.A., Mcdonough V.L., Doty S.M. (2018). Invasive Lionfish (*Pterois volitans/miles*) feeding ecology in Biscayne National Park, Florida, USA. Biol. Invasions.

[18] Huang Z., Yao H., Wang M., Liu Y., Chen M., Zhong M., Qiao J. (2024). Tracking the Effects of Mangrove Changes and *Spartina alterniflora* Invasion on Soil Carbon Storage: A Case Study of the Beibu Gulf of Guangxi, China. Land.

[19] Haisheng L.I., Ting Z., Canxiong W.U., Jingjian L., Meixia O. (2018). The Current Status and Conservation of Mangrove Resources in Tantoucun, Nansha, Guangzhou. J. Guangdong Univ. Educ..

[20] Ren H., Guo Q., Liu H., Li J., Zhang Q., Xu H., Xu F. (2014). Patterns of Alien Plant Invasion across Coastal Bay Areas in Southern China. J. Coast. Res..

[21] Ruiz G., Fofonoff P., Carlton J., Wonham M., Hines A. (2000). Invasion of Coastal Marine Communities in North America: Apparent Patterns, Processes, and Biases. Annu. Rev. Ecol. Syst.

[22] Isebor C.E., Ajayi T.O., Anyanwu A.O. (2003). The incidence of *Nypa fruticans* (Wurmb) and its impact on fisheries production in the Niger Delta mangrove ecosystem. Environ. Sci..

[23] Donnelly M.J., Green D.M., Walters L.J. (2008). Allelopathic effects of fruits of the Brazilian pepper *Schinus terebinthifolius* on growth, leaf production and biomass of seedlings of the red mangrove *Rhizophora mangle* and the black mangrove *Avicennia germinans*. J. Exp. Mar. Biol. Ecol..

[24] Zhang C., Guo J., Saveanu L., Martín P.R., Shi Z., Zhang J. (2023). Invasiveness of Pomacea canaliculata: The Differences in Life History Traits of Snail Populations from Invaded and Native Areas. Agronomy.

[25] O’Neil C.M., Guo Y., Pierre S., Boughton E.H., Qiu J. (2023). Invasive snails alter multiple ecosystem functions in subtropical wetlands. Sci. Total Environ..

[26] Montanari A., Bergamini G., Ferrari A., Ferri A., Nasi M., Simonini R., Malagoli D. (2024). The immune response of the invasive golden apple snail to a nematode-based molluscicide involves different organs. Biology.

[27] Celis-Ramírez M., Quintero-Angel M., Varela-M R.E. (2022). Control of invasive alien species: The Giant African snail (*Lissachatina fulica*) a difficult urban public management challenge. J Environ. Manag..

[28] Qin Z., Yang M., Zhang J.-E., Deng Z. (2020). Effects of salinity on survival, growth and reproduction of the invasive aquatic snail *Pomacea canaliculata* (Gastropoda: Ampullariidae). Hydrobiologia.

[29] Liu J., Chen Z., Li Y., Chen D., He Y., Zhao B., Liao Y., Guo J. (2023). Potential detritivorous diet of the invasive apple snail (*Pomacea canaliculata* Lamarck, 1822) in mangroves: The relationship between feeding indicators and chemical characteristics of decaying leaf litter. J. Mar. Sci. Eng..

[30] Wei M., Mao W., Wenqing W., Yi L., Liuqing L., Chaoyi T. (2018). Biodiversity of mangrove mollusks in the west coast of Hainan Island, China. Biodiv. Sci..

[31] Collado G.A., Vidal M.A., Torres-Diaz C., Cabrera F.J., Araya J.F., Darrigran G. (2020). Morphological and molecular identification of the invasive freshwater snail *Physa acuta* (Gastropoda: Physidae) into Llanquihue Lake, Chilean Patagonia. An. Da Acad. Bras. De Ciências.

[32] Liu P., Zhao B., Zhang J., Qin Z., Zhang C., Jing Q., Guo J. (2022). Responses of survival, growth, and feeding of the invasive Golden Apple Snail (*Pomacea canaliculata*) to salinity stress. Freshw. Sci..

[33] Eshra E.-S., Abdelgalil G., Gad A. (2024). Efficiency evaluation of two chemical pesticides and a biocide for controlling Eobania vermiculata snails infesting Guava orchards. Alex. J. Agric. Sci..

[34] Guo J., Zhang S., Zeng J., Chen Y., Guo Y., Liu J., He A. (2023). Molluscicidal activity of Nicotiana tabacum extracts on the invasive snail *Pomacea canaliculata*. Sci. Rep..

[35] Li S., Zou Z. (2019). Toxicity of Chimonanthus nitens flower extracts to the golden apple snail, *Pomacea canaliculata*. Pestic. Biochem. Phys..

[36] Cui L., Berger U., Cao M., Zhang Y., He J., Pan L., Jiang J. (2023). Conservation and Restoration of Mangroves in Response to Invasion of *Spartina alterniflora* Based on the MaxEnt Model: A Case Study in China. Forests.

[37] Mitra S., Naskar N., Lahiri S., Chaudhuri P. (2023). A study on phytochemical profiling of *Avicennia marina* mangrove leaves collected from Indian Sundarbans. Sustain. Chem. Environ..

[38] Sandrawati N., Ningsih W., Layla R., Eka Putra A., Ismed F., Ekawati Tallei T., Handayani D. (2023). Endophytic Fungi from Mangrove Plant *Acanthus ilicifolius* L.: Antimicrobial, Anticancer, and Species Determination. Trends Sci..

[39] Sarkar P., Ahnaf T.R., Rouf R., Shilpi J.A., Uddin S.J. (2024). A Review on Bioactive Phytochemical Constituents and Pharmacological Activities of *Aegiceras corniculatum*: A Pharmaceutically Important Mangrove Plant. J. Chem..

[40] Limbago J., Sosas J., Gente A., Maderse, Rocamora M., Gomez D.K. (2021). Antibacterial effects of mangrove ethanolic leaf extract against zoonotic fish pathogen *Salmonella arizonae*. J. Fish..

[41] Debnath S.L., Kundu P., Golder M., Biswas B., Sadhu S.K. (2020). Phytochemical Characterization and Evaluation of Pharmacological Activities of Leaves of a Mangrove Plant Species-*Aegiceras corniculatum* (L.). Trop. J. Nat. Prod. Res. (TJNPR).

[42] Abubakar A.R., Haque M. (2020). Preparation of Medicinal Plants: Basic Extraction and Fractionation Procedures for Experimental Purposes. J. Pharm. Bioallied Sci..

[43] Fayed M.A.A., Bakr R.O., Yosri N., Khalifa S.A.M., El-Seedi H.R., Hamdan D.I., Refaey M.S. (2023). Chemical profiling and cytotoxic potential of the n-butanol fraction of *Tamarix nilotica* flowers. BMC Complement. Med. Ther..

[44] Nguyen T.T., Lam M.K., Uemura Y., Mansor N., Lim J.W., Show P.L., Tan I.S., Lim S. (2020). High biodiesel yield from wet microalgae paste via in-situ transesterification: Effect of reaction parameters towards the selectivity of fatty acid esters. Fuel.

[45] Wu Y., Chen H., Wang B., Xu J., Li J., Ying G., Chen K. (2024). Extraction of *Ampelopsis japonica* polysaccharides using p-toluenesulfonic acid assisted n-butanol three-phase partitioning: Physicochemical, rheological characterization and antioxidant activity. Int. J. Biol. Macromol..

[46] Abdullah N.S., Aziz N.A., Mailon R. (2017). Molluscicidal activity of *Entada rheedii* stem bark methanolic extract against paddy pest *Pomacea canaliculata* (golden apple snail). Malays. J. Anal. Sci..

[47] Shen X., Wang Z., Liu L., Zou Z. (2018). Molluscicidal activity of *Solidago canadensis* L. extracts on the snail *Pomacea canaliculata* Lam. Pestic. Biochem. Phys..

[48] Ravi S., Young T., Macinnis-Ng C., Nyugen T.V., Duxbury M., Alfaro A.C., Leuzinger S. (2020). Untargeted metabolomics in halophytes: The role of different metabolites in New Zealand mangroves under multi-factorial abiotic stress conditions. Env. Exp. Bot..

[49] Ballada K.A., Baoanan Z.G. (2023). Molluscicidal properties of wild sunflower (*Tithonia diversifolia*) leaf extract fractions against invasive golden apple snail (*Pomacea canaliculata*). Environ. Dev. Sustain..

[50] Kamari A., Jumadi J., Sapie S.R., Wong S.T.S. (2023). Molluscicidal activity of *Tinospora crispa* Stem extracts against *Pomacea canaliculata*. AIP Conf. Proc..

[51] Chuong Nguyen T.H., Kim Lien G.T., Yen P.H., Ho T.-T., Thuy Van D.T., Van Kiem P., Hung N.H., Kuo P.-C., Setzer W.N.J.N.P.C. (2022). Molluscicidal activity of compounds from the roots of *Aralia armata* against the golden apple snail (*Pomacea canaliculata*). Nat. Prod. Commun..

[52] He D., Li C., Su Q., Lin Y., Zou Z. (2024). Screening the Efficacy and Safety of Molluscicides from Three Leaf Extracts of *Chimonanthus* against the Invasive Apple Snail, *Pomacea canaliculata*. Molecules.

[53] Dai L., Wang W., Dong X., Hu R., Nan X. (2011). Molluscicidal activity of cardiac glycosides from *Nerium indicum* against *Pomacea canaliculata* and its implications for the mechanisms of toxicity. Env. Toxicol. Phar..

[54] Wang X., Deng K., Zhang P., Chen Q., Magnuson J.T., Qiu W., Zhou Y. (2024). Microplastic-mediated new mechanism of liver damage: From the perspective of the gut-liver axis. Sci. Total Environ..

[55] Lodge M., Dykes R., Kennedy A. (2024). Regulation of Fructose Metabolism in Nonalcoholic Fatty Liver Disease. Biomolecules.

[56] Zhang L., Zou Z. (2020). Molluscicidal activity of fatty acids in the kernel of *Chimonanthus praecox cv*. Luteus against the golden apple snail *Pomacea canaliculata*. Pestic. Biochem. Phys..

[57] Jiang C., Storey K.B., Yang H., Sun L. (2023). Aestivation in nature: Physiological strategies and evolutionary adaptations in hypometabolic states. Int. J. Mol. Sci..

[58] Carmo de Carvalho e Martins M.d., Martins, da Silva Santos Oliveira A.S., da Silva L.A.A., Primo M.G.S., de Carvalho Lira V.B. (2022). Biological indicators of oxidative stress [malondialdehyde, catalase, glutathione peroxidase, and superoxide dismutase] and their application in nutrition. Biomarkers in Nutrition.

[59] Mas-Bargues C., Escriva C., Dromant M., Borras C., Vina J. (2021). Lipid peroxidation as measured by chromatographic determination of malondialdehyde. Human plasma reference values in health and disease. Arch. Biochem. Biophys..

[60] Richbart S.D., Merritt J.C., Nolan N.A., Dasgupta P. (2021). Acetylcholinesterase and human cancers. Adv. Cancer Res..

[61] Uluturhan E., Darılmaz E., Kontas A., Bilgin M., Alyuruk H., Altay O., Sevgi S. (2019). Seasonal variations of multi-biomarker responses to metals and pesticides pollution in *M. galloprovincialis* and *T. decussatus from* Homa Lagoon, Eastern Aegean Sea. Mar. Pollut. Bull..

[62] Sallam A., Mira A., Ashour A., Shimizu K. (2016). Acetylcholine esterase inhibitors and melanin synthesis inhibitors from Salvia officinalis. Phytomedicine.

[63] Wang L., Wu N., Zhang Y., Wang G., Pu S., Guan T., Zhu C., Wang H., Li J. (2022). Effects of copper on non-specific immunity and antioxidant in the oriental river prawn (*Macrobrachium nipponense*). Ecotoxicol. Environ. Saf..

[64] Qiu M., Wang Y., Wang X., Sun L., Ye R., Xu D., Dai Z., Liu Y., Bi S., Yao Y. (2016). Effects of T-2 toxin on growth, immune function and hepatopancreas microstructure of shrimp (*Litopenaeus vannamei*). Aquaculture.

[65] Yang Y., Sun Q. (2015). Analysis on main chemical components in oncomelania-inhibited extracts from leaf of *Sapium sebiferum*. J. Plant Res. Environ..

[66] Alnahdi A., John A., Raza H. (2019). Augmentation of glucotoxicity, oxidative stress, apoptosis and mitochondrial dysfunction in HepG2 cells by palmitic acid. Nutrients.

[67] Hsu J.-Y., Lin H.-H., Chyau C.-C., Wang Z.-H., Chen J.-H. (2021). Aqueous extract of pepino leaves ameliorates palmitic acid-induced hepatocellular lipotoxicity via inhibition of endoplasmic reticulum stress and apoptosis. Antioxidants.

[68] Sharma G., Parihar A., Parihar P., Parihar M.S. (2019). Downregulation of sirtuin 3 by palmitic acid increases the oxidative stress, impairment of mitochondrial function, and apoptosis in liver cells. J. Biochem. Mol. Toxicol..

[69] Banerjee P., Gaddam N., Chandler V., Chakraborty S. (2023). Oxidative Stress–Induced Liver Damage and Remodeling of the Liver Vasculature. Am. J. Pathol..

[70] Nansha District Meteorological Bureau, G.C. Nansha District Government of Guangzhou. Overview of Climate in Nansha District. https://www.gzns.gov.cn/nssj/zyhj/content/post_9365100.html.

[71] Barkat A., Khan B., Naveed A., Rasul A., Mahmood T., Muhammad H., Khan H.M.s., Iqbal M., Murtaza G. (2012). Investigation of the effects of extraction solvent/technique on the antioxidant activity of *Cassia fistula* L.. J. Med. Plants Res..

[72] Liu J., Zhao B., Li Y., Deng X., Qiao Y., Xu J., Xu S. (2022). Palatability of mangrove leaves to invasive apple snails: The relation between feeding electivity and multiple plant characteristics. Aquat. Invasions.

[73] Santos J.A.A.d., Tomassini T.C.B., Xavier D.C.D., Ribeiro I.M., Silva M.T.G.d., Filho Z.B.d.M. (2003). Molluscicidal activity of Physalis angulata L. extracts and fractions on *Biomphalaria tenagophila* (d’Orbigny, 1835) under laboratory conditions. Memórias Do Inst. Oswaldo Cruz.

[74] Bae M.-J., Chon T.-S., Park Y.-S. (2015). Modeling behavior control of golden apple snails at different temperatures. Ecol. Model..

[75] Zheng Y., Yang Z., Luo J., Zhang Y., Jiang N., Khattak W.A. (2023). Transcriptome analysis of sugar and acid metabolism in young tomato fruits under high temperature and nitrogen fertilizer influence. Front. Plant Sci..

[76] Li X., He T., Mao Y., Mao J., Lai X., Tu H., Zhou Y., Sha R. (2023). Changes in physicochemical properties, metabolites and antioxidant activity of edible grass during spontaneous fermentation. Fermentation.

[77] Li X., Li Y., Gao J., Mi S., Mao K., Zhang T., Wang X., Sang Y. (2023). Chemical composition of naturally-fermented mixed fruit product and in vitro bioactivities. LWT.

[78] Ngernsoungnern P., Rungsawang P., Janthaweera A., Duangsuwan P., Saowakon N., Sritangos P., Ngernsoungnern A. (2024). Ultrastructural study of neuronal cells and localization of ghrelin-like peptide and its receptor in the ganglia of the golden apple snail (*Pomacea canaliculata*). Tissue Cell.

[79] Wulandari A.P., Nafisa Z.K., Herlina T., Maharani R., Darmawan G., Parikesit A.A., Zainul R. (2024). Metabolite profiling of potential bioactive fractions from ethanol extract of Boehmeria nivea flowers by GC–MS/MS analysis. Phytomed. Plus.

[80] Wang L., Jing S., Wang S., Xing Z., Qu J., Wang X. (2024). Chemical Composition, Larvicidal and Ovicidal Activities, and Enzyme Inhibition Capacity of *Thymus serpyllum* Essential Oils Against *Spodoptera litura* (Fabricius). Plants.

[81] Hemalatha D., Rangasamy B., Nataraj B., Ramesh M. (2019). Assessment of triclosan impact on enzymatic biomarkers in an Indian major carp, *Catla catla*. J. Basic Appl. Zool..

