# Supplementary material for: Mangrove Against Invasive Snails: Aegiceras corniculatum Shows a Molluscicidal Effect on Exotic Apple Snails (Pomacea canaliculata) in Mangroves
Source: Plants (Basel). 2025 Mar 6;14(5):823. doi: 10.3390/plants14050823 (PMC11902146; doi:10.3390/plants14050823)
Supplement: Supplementary file 1 [file plants-14-00823-s001.zip › plants-3457985-supplementary.pdf]

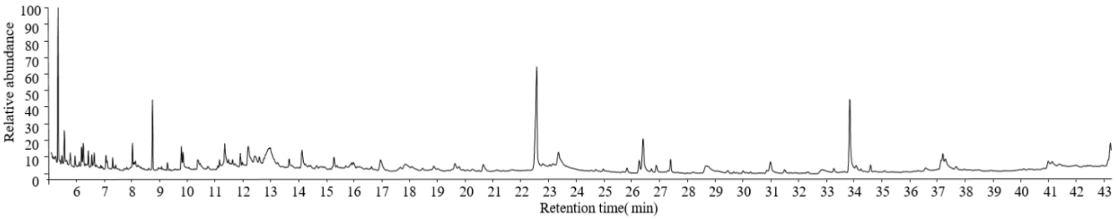

Figure S1. Total ion current diagram of n-butyl alcohol extract of *Aegiceras corniculatum*.

Table S1. Invasive organisms reported in mangrove wetlands

No	Invasive species	Order	Family	Native Range	Recorded invaded site	Type(plant/animal)	Reference
1	Teak defoliator moth (*Hyblaea puera* Cramer, 1777)	Lepidoptera	Hyblaeidae	Southern and South East Asia	Mangroves at the mouth of the Mamanguape River, Paraiba State, Brazil	animal	[67]
2	European shore crabs (*Carcinus maenas* Linnaeus, 1758)	Decapoda	Carcinidae	Northeast Atlantic and Baltic Seas, European and North African coasts	mangroves on the south coast of New South Wales (NSW) Australia	animal	[68]
3	Portunid crabs (*Charybdis hellerii* A. Milne-Edwards, 1861)	Decapoda	Portunidae	Indo-Western Pacific Region	Rhizophora mangle Linnaeus	animal	[69]
4	Green mussels (*Perna viridis* L., 1758)	Mytilida	Mytilidae	Indo-Pacific region	La Restinga Lagoon (Margarita Island, Venezuela)	animal	[70]
5	Cuban tree frogs (*Osteopilus septentrionalis* A.M.C. Duméril & Bibron, 1841)	Anura	Hylidae	Cuba, Bahamas	mangrove habitats in South Florida	animal	[71]
6	Prawns	Decapoda	Palaemonidae	South East Asia and	mangrove coasts of	animal	[72]

	(*Macrobrachium rosenbergii* De Man, 1876)			South Asia	northern Brazil		
7	River mangrove cichlids (*Tilapia mariae* Boulenger, 1899)	Perciformes	Cichlidae	West and Central Africa	northeast Queensland Australia	animal	[73]
8	Green iguanas (*Iguana iguana* L., 1758)	Squamata	Iguanidae	Central and South America, Lesser Antilles	mangrove habitat in Puerto Rico	animal	[74]
9	Lionfishes (*Pterois miles* J. W. Bennett, 1828)	Scorpaeniformes	Scorpaenidae	Indo-Pacific region	mangroves of Biscayne National Park, Florida, USA	animal	[75]
10	Pacific oyster (*Crassostrea gigas* Thunberg, 1793)	Ostreoida	Ostreidae	Northwest Pacific and Sea of Japan	New South Wales, Australia	animal	[70]
11	Smooth Cordgrass (*Spartina alterniflora* Loisel., 1807)	Poales	Poaceae	Atlantic coast of North America	Dandou Sea area in the Beibu Gulf of Guangxi	plant	[76]
12	Guatemalan Daisy (*Mikania micrantha*	Asterales	Asteraceae	Tropical South America and Central	Tantoucun, Nansha, Guangzhou	plant	[77]

	Kunth, 1818)			America			
13	Three-lobed Wedelia (*Wedelia trilobata* (L.) Park, 1928)	Asterales	Asteraceae	South-central region of tropical America	Shenzhen Bay and Leizhou Bay, China	plant	[78]
14	Morning Glory (*Ipomoea purpurea* (L.) Roth, 1832)	Solanales	Convolvulaceae	Colombia	Shenzhen Bay and Leizhou Bay, China	plant	[78]
15	Torpedo Grass (*Panicum repens* L., 1753)	Poales	Poaceae	Tropical and subtropical regions worldwide	Shenzhen Bay and Leizhou Bay, China	plant	[78]
16	Beach She-oak (*Casuarina equisetifolia* L., 1753)	Fagales	Casuarinaceae	Tropical and subtropical regions of Australia	Florida, USA	plant	[79]
17	Nipa Palm (*Nypa fruticans* Wurmb, 1780)	Arecales	Arecaceae	The coastlines and estuarine habitats of the Indian and	Niger Delta	plant	[80]

				Pacific Oceans			
18	Brazilian Peppertree (*Schinus terebinthifolius* Raddi, 1820)	Sapindales	Anacardiaceae	Brazil, Paraguay and Argentina	Florida, USA	plant	[81]

Table S2. Concentrations of mangrove extracts used in the molluscicidal test.

Extracts	Concentration (mg/L)				
Ethanol	0	20	25	30	35
Petroleum ether	0	100	120	140	160
Ethyl acetate	0	200	400	600	800
N-butanol	0	10	15	20	25
